# Evidence for a Bifactor Structure of the Caregiving Questionnaire with Individuals Involved in Different and Same-Sex Couple Relationships

**DOI:** 10.3390/ijerph17249306

**Published:** 2020-12-12

**Authors:** Mónica Guzmán-González, Carlos Calderón, Carol Murray, Diego Henríquez

**Affiliations:** School of Psychology, Universidad Católica del Norte, Avenida Angamos 0610, Antofagasta 1240000, Chile; ccalderon@ucn.cl (C.C.); carol.murray@alumnos.ucn.cl (C.M.); diego.henriquez01@alumnos.ucn.cl (D.H.)

**Keywords:** caregiving, bifactor models, couple relationships, attachment

## Abstract

Despite the Caregiving Questionnaire (CQ) being a widely used measure for the study of caregiving behavior in the context of romantic relationships, to date, few studies have focused on empirically evaluating its underlying theoretical structure. The aim of this study was to examine the factorial structure and equivalence across sex and sexual orientation of this instrument. A sample of 912 Chilean individuals currently involved in a couple relationship completed the Caregiving Questionnaire and the Experiences in Close Relationship Scale. After comparing various traditional Confirmatory Factor Analysis (CFA)models, the results provide support for a multidimensional and hierarchical nature of a brief 16-items version of the CQ. More specifically, the analyses supported a bifactor-CFA solution composed of two global factors and four specific factors, suggesting that they add information to the caregiving construct in the context of couple relationships. Additionally, the scale showed measurement invariance across sex and sexual orientation. Finally, significant associations were found between CQ scores with measures of romantic attachment in the expected directions. Theoretical implications about the nature of the caregiving system are discussed.

## 1. Introduction

The study of romantic relationships from the perspective of attachment theory has provided a framework for understanding love bonds between adults [1]. This theory suggests that romantic love during adulthood is based on three behavioral systems: the attachment system, which refers to the search for comfort and security, maintaining closeness with the partner; the caregiving system, which refers to the response and provision of support towards the needs of the partners seeking support; and the sexual/reproductive system, which encourages sexual expression, desire, and associated emotions within the couple [2].

The attachment and caregiving systems fulfill complementary functions in romantic relationships [3]. The first is activated when someone feels danger or under threat [4]. This triggers a search for closeness with the partner, in order for them to provide security and protection [5]. Meanwhile, the caregiving system is responsible for satisfying the said need for security in the other person through the provision of a safe haven that offers comfort and protection, as well as a secure base that serves as a source of encouragement for personal development over the environment [6,7]. The interaction between the attachment and caregiving systems allows both partners to give or receive support in situations of need [8], and their proper functioning translates into the continuance of satisfactory emotional bonds [9].

For the caregiving system to work properly, the caregiver requires empathy and sensitivity to connect with the needs of the other person, as well as the ability to respond in order to meet the requirements of their partner [7]. When these conditions are met, the partner receiving care can regulate his or her emotions and reduce discomfort, increasing safety, well-being, and the ability to face problems and/or challenges [10]. For their part, whoever provides care experiences satisfaction by contributing to their partner’s well-being, reinforcing feelings of altruism and empathy [11].

### 1.1. Attachment and Caregiving

From the framework of attachment theory, it has been proposed that the quality of early bonding experiences with significant figures translates into the development of representations called internal working models of the self and others, that shape the way in which care is sought and provided in relationships with others [2,12]. Achieving an experience of safety, that is, confidence that others will be available and responsive in times of need facilitates the correct functioning of the caregiving system. It has been found that people with a more secure attachment are warmer and more sensitive when responding to the needs of their partner, and are inclined towards providing empathetic and caring responses towards others [13,14].

Instead, when this experience of security is not reached, negative self-representations or of others develop, which can be expressed in two forms of insecurity: attachment anxiety and attachment avoidance [11]. Attachment anxiety refers to constant concern about potential abandonment on the part of the partner, marked emotional dependence and a continuous search for approval and love, based on a negative model of the self. In turn, attachment avoidance refers to distress and discomfort with emotional closeness and an excessive emphasis on self-sufficiency and independence, characteristics that are based on a negative image of others [1]. According to Mikulincer and Shaver [11], attachment insecurity is associated with how care is provided in romantic relationships, either due to hyperarousal or deactivation of the caregiving system. Among those with high levels of attachment anxiety, hyperactivation of the care system is observed, which leads them to be intrusive and hyper-vigilant, assuming a leading role in solving problems, or displaying exaggerated reactions that control or limit the partner’s actions. Accordingly, it has been reported that people with greater attachment anxiety, although seeking intimacy with others, tend to provide ineffective care, given that the motivation for caregiving would respond more to self-centered needs: either to reduce the distress generated by the partner’s vulnerability, or the need to be recognized and validated [11,15,16].

On the other hand, among those who show higher levels of attachment avoidance, the deactivation of the caregiving system is observed, which implies a low motivation to respond to the partner’s needs, a tendency to withdraw care, insufficient empathy to understand the requirements of the other person, and the adoption of an evasive or controlling stance [11,17]. Fittingly, it has been shown that people with high attachment avoidance display less compassion and caring behaviors towards their partners, given that they dislike exposure to anxiety and tend to perceive their partner’s behavior as dependent [18,19]. In short, both forms of attachment insecurity interfere with the ability to provide sensitive and responsive care to the needs of others. 

### 1.2. Measuring Caregiving in Couple Relationships

Given the relevance of the caregiving system to understanding relationships between adults, various scales have been created that address people’s strategies when providing care to others. Following the tradition of attachment theorists, Kunce and Shaver [17] wondered about individual differences in the caregiving system and how these operate in the context of adult romantic relationships. Based on the literature on parental caregiving towards children, and contrasting this information with open interviews aimed at caregiving in couples, these authors designed a questionnaire with seven dimensions that reflected the caregiver’s behavior in adult romantic relationships, called the Caregiving Questionnaire (CQ). 

Of the seven dimensions, four (sensitivity, acceptance, cooperation, and accessibility) had already been identified by Ainsworth, Blehar, Waters, and Wall [20], while the other three (physical contact, affective expression, and compulsive caregiving) were included through frequent use in studies on romantic ties [17].

However, after a review of the items and revision of this initial structure, the authors developed a proposal for the scale that distinguishes four dimensions that represent caregiving in romantic relationships. These dimensions were operationalized as follows: (1) proximity, which reflects an individual’s capacity to provide their partner with physical and psychological accessibility; (2) sensitivity, which refers to the ability to quickly perceive and accurately interpret the partner’s needs, feelings and attachment signals; (3) control, which measures the difficulty to respect the partner’s space and autonomy to solve problems on their own; (4) compulsive caregiving, which assesses the individual’s over-involvement in their partner’s problems and difficulties. Kunce and Shaver [17] reported that the questionnaire scores obtained reliability coefficients of 0.83, 0.83, 0.87 and 0.80, respectively. Likewise, they reported the reliability of the instrument through evidence of temporal stability, re-surveying 30 individuals one month after the first application. The test–retest results for each dimension were: proximity, *r* = 0.77; sensitivity, *r* = 0.78; control, *r* = 0.88; compulsive caregiving, *r* = 0.81. 

The CQ scale has been widely used in previous studies [15,16,18,21,22,23,24,25], with heterosexual couples who were dating [18], married young couples [23,24], and older couples [16]. Studies in the field have shown that caregiving dimensions are not correlated with age or relationship length [21], which is in line with the Bowlby conceptualization of caregiving as a universal system [3].

Despite its common use in couple relationships research, findings that support and confirm the factorial structure proposed in the original study of the CQ are unknown. Although there is some evidence that supports the presence of the four caregiving factors, these studies have been carried out using Exploratory Factor Analysis (EFA) [21], which generates factorial indeterminacy problems [26], or Principal Component Analysis (PCA) [17], whose use is discouraged for measurement instruments’ internal structure evidence assessment [27,28,29,30,31,32]. Furthermore, and regarding the association between the caregiving dimensions, studies offer contradictory evidence on the relationships between them. While in the original study, Kunce and Shaver [17] report moderate correlations between the dimensions, Bouaziz et al. [21] present evidence that supports an orthogonal factorial structure. Incidentally, several studies show the independence of the compulsive caregiving dimension [33], even to the point of not considering it as part of the caregiving construct or treating it separately [16,34,35]. Added to this is the fact that although the comparison between groups is a frequent practice in research [e.g., 15], there is little evidence about the equivalence of the CQ scale based on sex and type of romantic relationship (different and same-sex), which is an essential requirement for the comparability of measurements [36].

All this information makes an examination into the factorial structure of the CQ advisable. The lack of consistency of previous findings regarding the internal structure of the scale and the association between its dimensions could be due to the existence of an underlying structure that is more complex than that represented by a first-order factorial model. For example, the possibility of a more general orientation towards oneself and/or towards others, beyond the forms of caregiving in a specific relationship, as in the case of a romantic partnership. As Feeney and Hohaus [35] suggest, vis à vis the partner’s demand for care, people must additionally deal with more self-centered aspects, which are marked by the difficulty of putting aside their own needs regarding the relationship in order to respond to the care needs demanded by the partner.

This would mean that, alongside the existence of the caregiving dimensions, which are oriented towards the current partner, are dimensions related to the caregiver’s own capacities or difficulties in providing care in a broader sense. Hence, these can reflect more general patterns, which could be independent of the dimensions oriented towards a specific partner. In support of this proposition, attachment theory suggests that the self and others’ internal working models are formed early in life based on repeated interactions with significant others. These models shape how a person provides care across different contexts [3]. Dealing with another person´s suffering and needs requires empathy, feelings of genuine compassion, as well as directing attention to another´s needs. This is possible when the person has experienced effective care from her/his attachment figures. These experiences provide a model to follow when the person comes to assume the caregiver’s role [7,37]. Instead, when the internal working models are negative, care provision is more self-centered, be it to reduce the person´s discomfort or satisfy unmet needs for closeness and acceptance [11]. 

Faced with the hypothesis of the existence of this complex structure, bifactor models provide the possibility of explicitly modelling the general underlying factors that are independent of the specific substantive factors. Bifactor models allow for separating the variance attributable to specific factors from the variance attributable to general factors, allowing the simultaneous estimation of the direct relationships between the items and the specific and global factors [38]. Thus, bifactor models evaluate whether a global construct, reflected through a general factor, exists as a unitary dimension underlying the responses to all items, coexisting with other specific factors, also reflected by the same items [39].

### 1.3. The Present Study

Based on the above, the purpose of this study is to examine the factorial structure of the CQ scale, providing evidence in order to contribute to the discussion about its underlying theoretical structure. Since previous studies have only considered the application of exploratory techniques, an analysis of the internal structure of the CQ scale is proposed using a confirmatory approach through Confirmatory Factor Analysis (CFA). The use of this technique not only allows us to contrast the four-factor structure proposed in the original study [17], but also makes it possible to compare it with more complex alternative structures, in this case, the bifactor models. In the bifactor models, the items saturate in general and specific factors simultaneously, as a way to solve structural ambiguity problems [40]. According to previous studies [41], this structural ambiguity may be due to the underlying orthogonal characteristics of the structure that cannot be recovered by conventional factorial models. This will enable us to evaluate hypotheses regarding the existence of one or more general factors that represent a dimension or dimensions of self-orientation and/or orientation towards others, which would be independent of the dimensions of caregiving in a romantic relationship. Next, we will examine whether the internal structure of the CQ scale is equivalent/invariant according to sex and sexual orientation, which are usually variables of interest in studies on romantic relationships [17,21,34]. Finally, and in order to add evidence of the scale’s construct validity, we will examine the association between caregiving factors and the dimensions of adult attachment: attachment anxiety and avoidance.

Based on what has been explained thus far, the hypotheses that guide the present study is that the CQ scores will show evidence of structural validity through a measurement model adjusted to four factors with moderate- to high-factor loadings (λ > 0.4) in each one of its items. Although there is no prior evidence that allows the formulation of specific hypotheses regarding the complexity of the factorial structure, it is possible to propose an exploratory hypothesis. Given the possible existence of a dimension of individual orientation, which is independent of the dimensions of caregiving, it is expected that this dimension will be represented in a bifactor structure, which will not present relationships with the specific caregiving factors. Secondly, we anticipate that the internal consistency of the version examined will be comparable with the original CQ scale. Third, we propose that the CQ scale will be invariant between men and women and between those who are in different and same-sex relationships. Finally, we hypothesize that the scores of the CQ’s proximity and sensitivity factors will show significant and inverse relationships with the scores of the dimensions of attachment anxiety and avoidance. In the same vein, we propose that the scores of control and compulsive caregiving factors of the CQ will show significant and direct relationships with attachment anxiety and avoidance scores.

The present research has several features that contribute to expanding past investigations in the field. To the best of our knowledge, this is the first study to formally explore if the theoretical structure of the CQ is supported by applying a more sophisticated and innovative approach through CFA techniques. Second, this study formally explores if the CQ scale has a similar functioning across men and women, which has not been addressed before, although sex comparison is frequently practiced in couple research. In the same vein, the equivalence of the CQ scale has not been investigated in the context of individuals involved in a same-sex or different-sex couple; even though similar results may be expected, examining this aspect is needed to have psychometrically sound measures, suitable for specific populations. Testing the equivalence of the measures is an essential step to make valid comparisons between groups [42], which has not been addressed in previous studies. Finally, the research involves a large sample of individuals involved in a couple relationship, who varied in terms of the age and length of their relationship. 

## 2. Materials and Methods

### 2.1. Design and Participants

The study was of an instrumental nature [43]. The choice of participants was made through a convenience sampling, organized according to quotas by age, sex, and relationship (different or same-sex). The inclusion criteria were being over 18 years of age, and being involved in a relationship with a romantic partner for at least three months.

Of the 1146 individuals who contacted the research team or accessed the link to participate, 1079 (94.2%) met the inclusion criteria, and 67 were excluded due to exclusion criteria (age, relationship length, or relationship status). Of these, 167 (15.4%) returned incomplete questionnaires with missing key variables. Thus, the final sample was composed of 912 participants, of which 490 were women (53.7%) and 422 were men (46.3%). A total of 64.4% (*n* = 587) of the participants indicated being heterosexual and having a partner of the opposite sex, while 35.6% (*n* = 325) indicated being homosexual, bisexual or pansexual, and having a same-sex partner. The average age of the participants was 31.44 years old (SD = 10.7). Regarding educational level, 2.8% of the participants had completed primary education or less, 51.6% had achieved secondary or technical studies, while 45.6% reported having attended higher education. In terms of the duration of the couples’ relationships, the average was 6.69 years (SD = 8.07, minimum 3 months, maximum 51 years, median = 3.79 years) and 50.7% (*n* = 462) reported cohabiting with their respective partners. The group of women was younger than the group of men, *t* (907) = 2.97, *p* = 0.003, *d* = 0.20, but no differences were detected in the relationship length, *t* (910) = −0.269, *p* = 0.788, *d* = 0.02. There was no association between sex and educational level, (χ^2^ (7) = 3.06, *p* = 0.88, V = 0.02).

### 2.2. Measurement Instruments

The *Caregiving Questionnaire* (CQ) [17] is a 32-item self-report scale on caregiving in romantic relationships. It comprises four theoretical dimensions: proximity, sensitivity, control, and compulsive caregiving. The response format is Likert type, with response options ranging from 1 (*nothing like me*) to 6 (*very similar to me*). High scores reflect high levels of closeness, sensitivity, non-controlling and non-compulsive caregiving. Participants were asked to answer each item, selecting the number that indicates how descriptive the statement was of them. The CQ was adapted to the Chilean context following a cross-translation procedure. The instrument was first translated into Spanish and then back-translated by two independent bilingual translators. Then, the instrument was reviewed by four expert judges (researchers with expertise in attachment theory and couple therapists) to evaluate the content equivalence of the translated version. Items that were not agreed upon were reformulated based on suggestions made by the judges and then piloted. The piloted participants had similar characteristics to the final sample. Hence, the generated version is conceptually and linguistically equivalent to the original CQ (See Appendix A).

The Spanish *Experiences in Close Relationships-12* (ECR-12) [44] is a validated self-report adult attachment scale for the Chilean population in a short version, consisting of 12 items, developed from the original 36-item version of Brennan et al. [45]. The ECR-12 assesses the two dimensions of adult attachment in romantic relationships: attachment anxiety (e.g., “I worry about being abandoned”) and attachment avoidance (e.g., “I don’t feel comfortable opening up to romantic partners”), which consist of six items each rated on a 7-point scale (1 = *strongly disagree* to 7 = *strongly agree*). Higher scores on the ECR represent more attachment insecurity. The ECR-12 has been shown to be a reliable measurement in different types of samples, with a Cronbach’s alpha that ranges between 0.72 and 0.83 for the attachment anxiety subscale and 0.78 to 0.89 for the attachment avoidance subscale. For this study, the values obtained from Cronbach’s alphas on the ECR-12 adult attachment scale for the attachment anxiety dimension was 0.71, and for attachment avoidance dimension 0.78.

### 2.3. Procedure

This study was part of a larger research aimed to explore the functioning in couple relationships of Chilean adults. The instruments and the procedure were known and approved by the ethics committee of the Universidad Católica del Norte, Chile. Participants were recruited through several means such as the snowball technique, advertisements on social networks, and through the intermediation of a previously trained research team.

Before applying the measurement instruments, the participants were provided with information about the nature of the study, the privacy of their data, and then were asked to sign an informed consent authorizing the use of their responses for research purposes. In the online administration case, informed consent was obtained by clicking the button “Yes, I consent to participate” displayed on the first page. The questionnaires were anonymous, applied in two modalities: paper and pencil, and online through the Survey Monkey platform. All participants completed the questionnaire individually. The duration of the questionnaire was approximately 30–40 min.

No differences were detected in the variables under study between paper-and-pencil and online administration of the questionnaires.

### 2.4. Data Analysis

For the internal structure evidence assessment of the CQ scale, CFA was used. In the first stage, the original factorial structure presented by Kunce and Shaver [17] was evaluated. Likewise, and to obtain a refined scale with better statistical adjustment, items with factor loadings lower than 0.40 were eliminated, following the strictest recommendations of previous works [46,47,48]. Values around 0.30 were not considered because they correspond to percentages of variance lower than 10% [49]. Subsequently, and to evaluate the hypotheses regarding the presence of a more complex structure, the original model results were compared with alternative bifactor models. To assess the reliability of the final model’s dimensions, the Composite Reliability (CR) and Average Mean Extracted (AVE) were obtained. Values over 0.50 for AVE and 0.70 for CR have been used as cut-off points [50,51]. In order to present more evidence of internal structure validity, two factorial invariance analyses were carried out using Multi-group Confirmatory Factor Analysis (MCFA) using sex and type of partner (same- and different-sex) as grouping variables. To assess the validity regarding the relationship with other variables, a correlation analysis was carried out between the scores of the CQ scale dimensions and the ECR-12 scale of attachment in adults.

For the data analysis, SPSS v25 (IBM, Armonk, NY, USA) and Mplus v7 software (Muthén & Muthén, Los Angeles, CA, USA) were used. For the estimation of the CFA and MCFA models, we used the Robust Weighted Least Squares (WLSMV) estimation method, which is robust with non-normal ordinal variables. Previous studies have shown that with categorical and ordinal data, the WLSMV estimation method obtains more appropriate rejection rates of the ꭓ^2^ test than with the ML method and the magnitude of the loadings is more precisely estimated [52]. For assessing the global fit of the models, we used the fit indices most utilized in research practice: the goodness of fit test through the χ^2^ statistic, the absolute adjustment indices RMSEA [53] and TLI [54,55], and Bentler’s CFI incremental adjustment index. As cut-off points for these statistics, the recommendations of Hu and Bentler [56] were followed.

## 3. Results

Results are presented in three sections. The first one reviews the results of the CFA analysis of the different models that were evaluated. The second reviews the results of the factor invariance analyses. Lastly, the third section presents the correlation analyses performed to assess the evidence regarding the relationship with other variables.

### 3.1. Confirmatory Factor Analysis

The fit indices of the original model by Kunce and Shaver [17] presented a poor fit to the data (χ^2^_458_ = 5387.883; *p* < *0*.05; CFI = 0.796; TLI = 0.779; RMSEA = 0.109). At the local level, it is observed that the model presents an excessive number of modification indices, some quite high (19 of them with values over 100), which shows the presence of a significant proportion of cross-loadings. Given the problem of indeterminacy of this solution, a filtering process was carried out, eliminating some items based on two criteria: factor loadings lower than 0.40 in their respective dimension and the presence of high cross-loadings with other dimensions. From this process, a reduced version of 16 items composed of four items per factor was obtained. This proposal was subjected to new analyses comparing different alternative solutions. The fit measures of these models are presented in Table 1.

The reduced version of the CQ, made up of the four factors proposed by the original study, presents unacceptable global fit indices (RMSEA < 0.08; CFI > 0.95; TLI *>* 0.95.)

The following two models correspond to alternative bifactor models with one and two general factors, respectively. The results show that the model fit improves considerably.

For the case of the bifactor model with a general factor, the fit indices present acceptable values, except for TLI (<0.95). Because of this, we specify a second bifactor model with two general factors. These general factors were identified by reviewing the items’ content and the direction of their loads with the general factor of the previous model. Two types of orientations in different directions were detected, which seem to be independent of caregiving dimensions: an orientation towards others, in which items are saturated with content that reflect interest and concern for another, and an orientation towards oneself, which implies self-centered behaviors. This factorial solution showed excellent global fit indicators (RMSEA = 0.051; CFI = 0.98; TLI = 0.97).

**Figure 1:**
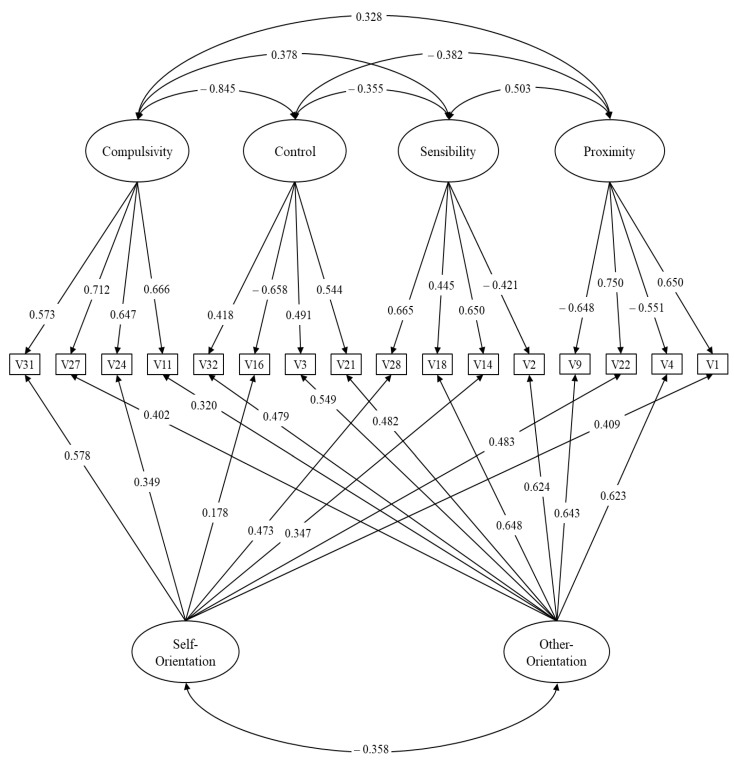
Path diagram of final model of the Caregiving Questionnaire.

Figure 1 presents the path diagram with the solution of the final model. Within the specific factors, the saturations presented factorial loads greater than 0.40, while the loadings of the general factors ranged from 0.17 to 0.64. Regarding correlations between factors: proximity had a high-magnitude relationship with sensitivity (0.50) and moderate-magnitude relationship with control (−0.38) and compulsion (0.32). On the other hand, the magnitudes of sensitivity were moderate with control (0.35) and compulsion (0.37) and, finally, compulsion and control; their magnitude ratio was high (−0.84).

### 3.2. Reliability

Descriptive statistics and reliability statistics of the dimensions obtained from the CFA are presented in Table 2. We have obtained Cronbach’s alpha coefficient, the Average Variance Extracted (AVE) and the Composite Reliability (CR) coefficient for the reliability analysis. No relevant associations were found between the caregiving dimensions with relationship length, sex, and age (see Appendix B).

### 3.3. Multigroup Confirmatory Factor Analysis

To assess the Equivalence/Invariance of measurement between different groups, two MCFA analyses were carried out, using sex and sexual orientation as grouping variables. The assessed model is the final model corresponding to the bifactor model with four specific caregiving factors and two general orientation factors. As is traditional, the analysis was performed in a hierarchical manner, studying the difference in fit between different nested models. These models correspond to the configural level (baseline model), the metric level, and the scalar level. Chen’s [57] recommendations were used to evaluate the difference in fit between the nested models (Δ_CFI_ < 0.01; Δ_TFI_ < 0.01; Δ_RMSEA_ *<* 0.01).

The results of the fit indices on the analysis by sex are presented in Table 3. As can be seen, the RMSEA, CFI and TLI fit indices do not show statistically significant differences in fit between any of the nested models. These results show that the scores for each of the dimensions of caregiving are equivalent between men and women.

Table 4 shows the fit indices for the MCFA using the type of partner as the grouping variable. Once again, the results show an excellent fit, without statistically significant differences being detected between the different nested models. These results show that the scores of the different dimensions are equivalent between people with same-sex partners and different-sex partners. In summary, the results of the factorial invariance analyses show that there is equivalence/measurement invariance of the factorial model both for men and women, and for people with different and same-sex partners.

### 3.4. Evidence Based on Relations to Other Variables

Table 5 shows the correlation coefficients between the specific and general dimensions of the CQ and attachment dimensions. We can see that there is a statistically significant and an inverse relationship between the proximity and sensitivity factors of the CQ with the attachment anxiety and avoidance factors in the Spanish ECR-12. For their part, the CQ’s control, and compulsive caregiving factors show significant and positive correlations with levels of attachment anxiety and avoidance. It is also possible to observe that the general factor orientation towards others shows an inverse correlation with attachment avoidance, and the self-orientation factor has a significant and positive correlation with attachment anxiety and avoidance. This indicates that close and sensitive caregiving is associated with a lower degree of attachment anxiety and avoidance, and that controlling and compulsive caregiving are associated with higher levels of attachment insecurity.

## 4. Discussion

This study aimed to review the factorial structure of the CQ scale for its use in a population of people involved in different- and same-sex romantic relationships. To our knowledge, the latent structure of the CQ scale has not been evaluated using an innovative approach such as a bifactor model, nor has its equivalence been tested across sex and between those involved in different and same-sex relationships.

The results indicate that the scores of a shortened version of the questionnaire would have a more complex factorial structure than that originally proposed by Kunce and Shaver [17]: the latent structure of the CQ scale is best delineated using a bifactor modeling approach where the subscale scores provide unique information above and beyond general factors. More specifically, the presence of four specific factors (proximity, sensitivity, control, and compulsion) and two general factors (orientation towards others and self-orientation) were identified, whose fit was superior to the other models that were tested.

In the case of the general factors, these could reflect global dispositions, which account for the individual differences in caregiving in relationships in general, beyond what is experienced in a particular relationship. On the one hand, orientation towards others could account for a global concern for others’ needs, postponing one’s own interests, implying a willingness to provide empathic care to others in times of need. For example, other-orientation can be characteristic of a secure script, in which the person can recognize others´ needs and respond empathically. On the other hand, self-orientation could reflect the lack of connection with others´ needs, based on a more self-centered focus, which can be characteristic of more insecure scripts.

In this sense, the caregiving behaviors that are deployed could rather reflect the need to reduce one’s own discomfort. This potential explanation is in line with the findings reported by Feeney and Collins´s work [18] regarding the existence of relatively altruistic and egoistic motives for helping or not helping, and with studies in the field of empathy and compassion. On the other hand, the specific factors would indeed account for the caregiving behaviors that are expressed in a particular relationship that can be influenced by its specific features, support recipients characteristics, among others.

The results corresponding to the model of four specific factors and two general factors suggest that it is necessary to take into account the disposition of the people evaluated, since caregiving strategies in romantic relationships may be affected by the orientation of the caregiver, be it an orientation centered on the needs of the partner or centered on one’s own needs. This could explain why an efficient or an optimal caregiver is not available at a certain time or vis-à-vis a particular partner, or how a caregiver who is characterized by using secondary strategies (hyperactivation or deactivation) may be genuinely interested in providing support and emotional containment for their partner, regardless of the difficulties involved in connecting with them.

This factorial structure with two general factors needs to be explored in future research aimed at seeking more evidence about the real nature of these general factors. For example, the relationship with other variables that have shown to be connected with the care provided could be examined, such as personality factors, mental health variables, self-esteem, among others [18]. However, these findings are consistent with Feeney and Collins’ [18] study that postulates that caregiving behavior is shaped by the individual difference factors that each partner brings with him or her into the relationships, as well as by the relationship´s characteristics.

Regarding the psychometric properties of the proposed model, these showed a good fit to the criteria recommended by the literature. The factor loadings were within those expected according to the hypotheses that were initially raised (λ > 0.4). Likewise, the multi-group results show that the proposed model is equivalent across sex and sexual orientation, which can be interpreted as CQ reflecting the same construct between men and women, and between individuals involved in different- and same-sex couple relationships. Hence, comparisons across groups based on these variables (sex and sexual orientation) can be implemented.

In terms of the CQ internal consistency, the alpha coefficient shows acceptable reliability, especially for the specific factors, considering that they have a reduced number of items. Regarding the AVE results, the values do not reach the minimum recommended criteria of 0.50. These results should be interpreted with caution since this index captures each item’s average explained variance in each factor. In a bifactor model, each item shares its explained variability with both specific and general factors. This is reflected in the CR results, which capture the proportion of common variance in relation to the error variance. The CR coefficient shows that, except for Control, all dimensions achieve values above 0.70. Overall, the results show that the dimensions obtained have acceptable reliability.

Furthermore, the CQ specific factors tended to correlate with constructs from attachment theory in the expected directions, providing additional data on the validity of this instrument. As anticipated, attachment anxiety was more strongly associated with controlling and compulsive caregiving, while attachment avoidance was more strongly connected with low proximity and sensitivity, findings that are in line with previous evidence [15,16,18,22]. These results can represent the tendency to become overly involved in the case of people with higher attachment anxiety, even though they experience concern for their partner´s welfare [7]. In the case of individuals with higher attachment avoidance, these associations could be explained in terms of their tendency to maintain an emotional distance that interferes with their responsive and empathic caregiving [14,58]. Attachment anxiety was also associated with lower levels of proximity and sensitivity; this finding is congruent with the previous literature, showing that the vulnerability to personal distress experienced by individuals that are more anxiously attached tends to hinder effective care [11,14,58]. For its part, the association detected between attachment avoidance with control and compulsive caregiving may be due to the domineering behavior detected among these individuals [11]. 

Besides, the correlations presented by the general factors and the attachment dimensions are also congruent with what could theoretically be expected from the attachment conceptual framework. Attachment avoidance was related to higher self-orientation and lower other-orientation, which is consistent with the lower empathic concern, and more self-focused motivations when helping others, based on a negative view of others. Attachment anxiety was associated with higher self-orientation only, consistent with the high personal distress and the desire for approval when helping based on the lack of self-confidence [11]. No association was detected with other-orientation, which can be the result of two different motives that coexist in people who exhibit more attachment anxiety: the concern for the other´s welfare, but, at the same time, the overinvolvement and the use of caregiving as a way of satisfying their own needs. Although the examination of the factorial structure of a scale that is widely used represents progress in providing evidence of its construct validity, the present study must be considered in light of certain limitations. First, even when the sample of the current study was relatively large, it was composed of a culturally similar group (Chilean people), so the extent to which the bifactor structure of the CQ can be generalized to other populations is a remaining question. Although a core element of caregiving conceptualization within attachment theory is its universality [3], the role of contextual and sociocultural variables is also relevant [59]. Future studies should validate the CQ bifactor model in a broader range of cultures in order to generalize these findings. Second, the non-probabilistic nature of the sample and the greater representation of people with medium and high educational levels limits the generalizability of the results. Besides this, the data were collected through a self-report method, which is weak in its permeability to social desirability. Moreover, like any study that uses self-report measures, our results may be affected by method common variance or any other response bias type. Thus, it is necessary to apply techniques that evaluate the existence of common variance that does not correspond to substantive variance. Certainly, more studies are necessary to evaluate this aspect. Finally, the test–retest reliability of the CQ was not determined because the assessment protocol was administered only once. Hence, further studies with this shortened version of the CQ should be conducted to overcome the study’s limitations that may further refine of the scale and expand these findings. 

Possible lines of future research include examining the psychometric robustness of this bifactor structure and its equivalence across other groups of interest, for example, with couples who are dating and married couples, considering that expectations of caregiving could be more salient in more committed relationships [35]. In the same line, invariance analyses as a function of age, relationship length, or between couples under more stressful situations (e.g., chronic illness or a major health crisis) compared to those facing daily life stressors could be of interest. Future work can also examine actor–partner effects of caregiving withing different- and same-sex couples. 

Our findings have potential implications for clinicians working with couples as well. The CQ is one of the most regularly utilized instruments to measure caregiving within couples, and this study contributes to having suitable instruments for the clinical context. In light of the present findings and if replicated in future studies, the bifactor model would provide a means for assessing general caregiving orientations and specific expressions of providing care within the relationship reflected in the model subdimensions. Professionals could also find it helpful to consider the impact of individual differences in caregiving orientations be in daily life or under stressful situations, in the design of their interventions. The examination of how caregiving is expressed in elderly couples who are more likely to require longer periods of caregiving could be of particular interest for health professionals, given that, usually, the partner is the main source of such care.

## 5. Conclusions

In summary, the results obtained support the existence of a bifactor structure of the abbreviated version of the CQ, whose fit is superior to those of an oblique model with adequate psychometric properties for use in people involved in a couple relationship with either different or the same sex. In this way, we have an instrument whose brevity makes it useful for application both in research and in clinical practice. Besides this, the possibility of its use in people who self-identify as homosexual is an opportunity to broaden the understanding of couple relationships in this population.

## Figures and Tables

**Table 1:** Goodness of fit indices of alternative model.

Models	*χ^2^*	*Gl*	*p*	RMSEA	CFI	TLI
Four-factor model	1935.258	98	<0.001	0.144	0.815	0.774
Four-factor one-bifactor model	463.301	82	<0.001	0.071	0.959	0.940
Four-factor two-bifactor model	270.633	81	<0.001	0.051	0.980	0.970

**Table 2:** Descriptive statistics and reliability of caregiving factors.

Caregiving Factors	M	SD	alfa	AVE	CR
Specific factors					
Compulsivity	2.812	1.098	0.714	0.424	0.811
Control	4.172	0.712	0.665	0.282	0.683
Sensibility	3.693	0.647	0.696	0.309	0.747
Proximity	3.564	0.545	0.748	0.427	0.862
General factors					
Self-orientation	3.861	0.538	0.725	0.294	0.863
Other-orientation	3.797	0.534	0.755	0.180	0.751

**Table 3:** Goodness of fit indices of nested model of factor invariance analysis according to sex.

Models	χ^2^	gl	*p*	RMSEA	CFI	TLI	Δχ^2^	Δgl	Δp
Configural	360.074	174	<0.001	0.049	0.980	0.973			
Metric	381.036	190	<0.001	0.047	0.980	0.975	43.555	16	0.001
Scalar	528.288	270	<0.001	0.046	0.973	0.976	175.054	80	<0.001

**Table 4:** Goodness of fit indices of nested model of factor invariance analysis according to sexual orientation.

Models	χ^2^	gl	*p*	RMSEA	CFI	TLI	Δχ^2^	Δgl	Δp
Configural	361.581	174	<0.001	0.049	0.980	0.973			
Metric	403.978	190	<0.001	0.050	0.978	0.972	52.207	16	<0.001
Scalar	450.625	270	<0.001	0.038	0.980	0.983	111.213	80	0.012

**Table 5:** Correlation of caregiving dimensions and attachment dimensions.

Caregiving Factors		Attachment Anxiety	Attachment Avoidance
Caregiving dimensions	Proximity	−0.11 *	−0.42 *
	Sensibility	−0.21 *	−0.28 *
	Control	0.31 *	0.22 *
	Compulsivity	0.32 *	0.22 *
Orientation dimensions	Other-Orientation	−0.01	−0.28 *
	Self-Orientation	0.13 *	0.36 *

Note: * *p* < 0.01.

## Appendix A

### Items Retained in the Final Version

1. (1) I sometimes push my partner away when s/he reaches out for a needed hug or kiss (A veces alejo a mi pareja cuando se me acerca para recibir un abrazo o beso que necesita) (R).
2. (2) I can always tell when my partner needs comforting, even when s/he doesn’t ask for it (Siempre noto cuando mi pareja necesita consuelo, incluso cuando no me lo pide).
3. (3) I always respect my partner’s ability to make his/her own decisions and solve his/her own problems (Siempre respeto la capacidad de mi pareja para tomar sus propias decisiones y resolver sus propios problemas) (R).
4. (4) When my partner cries or is distressed, my first impulse is to hold or touch him/her (Cuando mi pareja llora o está angustiada, mi primer impulso es darle un abrazo o tocarla).
5. (9) I feel comfortable holding my partner when s/he needs physical signs of support and reassurance (Me siento cómodo abrazando a mi pareja cuando necesita señales físicas de apoyo y seguridad).
6. (11) I tend to get overinvolved in my partner’s problems and difficulties (Tiendo a meterme demasiado en los problemas y dificultades de mi pareja).
7. (14) I sometimes miss the subtle signs that show how my partner is feeling (A veces no noto señales sutiles que muestran cómo se siente mi pareja) (R).
8. (16) I tend to be too domineering when trying to help my partner (Tiendo a ser demasiado dominante cuando trato de ayudar a mi pareja).
9. (18) I am very attentive to my partner’s nonverbal signals for help and support (Pongo mucha atención a las señales no verbales de mi pareja que me indican que necesita ayuda y apoyo).
10. (21) I can help my partner work out his/her problems without “taking control” (Puedo ayudar a mi pareja a resolver sus problemas sin “tomar el control”) (R).
11. (22) I sometimes draw away from my partner’s attempts to get a reassuring hug from me (A veces aparto a mi pareja cuando busca un abrazo para tranquilizarse) (R).
12. (24) I tend to take on my partner’s problems—and then feel burdened by them (Tiendo a hacerme cargo de los problemas de mi pareja, pero después siento que me agobian).
13. (27) I frequently get too “wrapped up” in my partner’s problems and needs (Suelo involucrarme demasiado en los problemas y necesidades de mi pareja)
14. (28) I sometimes “miss” or “misread” my partner’s signals for help and understanding (A veces no noto o leo mal las señales de mi pareja que me indican que necesita ayuda y comprensión) (R).
15. (31) I create problems by taking on my partner’s troubles as if they were my own (Cuando enfrento los problemas de mi pareja como si fueran míos, genero más dificultades).
16. (32) When helping my partner solve a problem, I am much more “cooperative” than “controlling” (Cuando ayudo a mi pareja a resolver un problema, soy mucho más cooperativo que controlador) (R).

Note. In brackets are indicated the original number of the items and the Spanish items. (R) refers to reversed items. Proximity: 1, 4, 5, 11; Sensitivity: 2, 7, 9, 14; Control: 3, 8, 10, 16; Compulsion: 6, 12, 13, 15

## Appendix B

**Table A1:** Comparison in CQ dimensions according to sex.

Caregiving Factors	Men		Women				
	M	SD	M	SD	t	*p*	d
Proximity	5.363	1.140	5.344	0.930	0.275	0.783	0.019
Sensibility	4.482	1.174	4.754	0.971	−3.824	<0.001	0.125
Control	2.360	0.929	2.471	0.994	−1.806	0.070	0.114
Compulsivity	2.753	0.929	2.859	1.182	−1.450	0.147	0.002
Self-orientation	2.571	0.795	2.680	0.904	−1.913	0.056	0.129
Other-orientation	4.923	1.042	5.049	0.833	−2.039	0.045	0.138

*p* < 0.01.

**Table A2:** Comparison in CQ dimensions according to relationship length (median).

Caregiving Factors	<3 Years		>3 Years				
	M	SD	M	SD	t	*p*	d
Proximity	5.372	1.120	5.320	0.927	0.758	0.449	0.048
Sensibility	4.623	1.146	4.631	1.007	−0.104	0.917	0.009
Control	2.384	0.954	2.494	0.981	−1.678	0.094	0.009
Compulsivity	2.823	1.114	2.821	1.080	0.026	0.979	0.000
Self-orientation	2.624	0.870	2.670	0.833	−0.761	0.447	0.058
Other-orientation	4.998	1.028	4.975	0.825	0.363	0.717	0.032

*p* < 0.01.

**Table A3:** Correlations between CQ dimensions, age and duration of couple’s relationships.

Caregiving Factors	Age	Relationship Length
Proximity	−0.091 **	−0.070 *
Sensibility	−0.055	0.005
Control	0.105 **	0.078 *
Compulsivity	0.025	0.034
Self-orientation	0.061	0.056
Other-orientation	−0.082 *	−0.036

Note: (**) *p* < 0.01; (*) *p* < 0.05.
